# Quantification of Urine and Plasma Levels of Extracellular Vesicles in a Cohort of Kidney Transplant Recipients and Chronic Kidney Disease Patients

**DOI:** 10.3390/ijms26083635

**Published:** 2025-04-11

**Authors:** Valentine Jacob, Quentin de Berny, François Brazier, Claire Presne, Julien Lion, Hakim Ouled-Haddou, Valérie Metzinger-Le Meuth, Gabriel Choukroun, Laurent Metzinger, Nicolas Guillaume

**Affiliations:** 1 HEMATIM UR-UPJV 4666, C.U.R.S, University of Picardie Jules Verne, F-80000 Amiens, France; valentine.jacob@aphp.fr (V.J.); lion.julien@chu-amiens.fr (J.L.); hakim.ouled-haddou@u-picardie.fr (H.O.-H.); valerie.metzinger@u-picardie.fr (V.M.-L.M.); laurent.metzinger@u-picardie.fr (L.M.); 2 Laboratory of Histocompatibility, Amiens University Hospital, F-80000 Amiens, France; 3 Department of Nephrology Dialysis Transplantation, Amiens University Hospital, F-80000 Amiens, France; deberny.quentin@chu-amiens.fr (Q.d.B.); brazier.francois@chu-amiens.fr (F.B.); presne.claire@chu-amiens.fr (C.P.); choukroun.gabriel@chu-amiens.fr (G.C.); 4 INSERM UMRS 1148, Laboratory for Vascular Translational Science (LVTS), UFR SMBH, University of Sorbonne Paris Nord, F-93000 Bobigny, France

**Keywords:** antibody-mediated rejection, extracellular vesicles, chronic kidney disease, kidney transplant recipients

## Abstract

Extracellular vesicles (EVs) have a key role in intercellular communication. We hypothesized that EVs are biomarkers of nephropathy or kidney allograft rejection. We screened patients with chronic kidney disease (CKD) and kidney transplant (KT) recipients. We measured the urine and plasma levels of total EVs overall and EV subpopulations (positive for podocalyxin, aquaporin-1, CD133, CD144, CD19, CD3, CD16, CD56, or CD41). We included 92 patients with CKD, 70 KT recipients, and 33 healthy volunteers. In CKD, the total urine EV concentration was correlated positively with the estimated glomerular filtration rate (eGFR), but none of the subpopulations was identified as a potential biomarker of nephropathy. Among the KT recipients, 30 had good allograft function and 40 had allograft disease (13 with antibody-mediated rejections (ABMR), 12 with T-cell-mediated rejection (TCMR), and 15 with allograft dysfunction). Patients with ABMR had low plasma levels of EVs derived from B-cells, T-cells, and endothelium (*p* = 0.003, 0.009, and 0.005, respectively). Patients with TCMR had a low urine level of EVs derived from endothelium (*p* = 0.05). EVs derived from B-cells, T-cells, and endothelium might be biomarkers of kidney allograft rejection. However, we did not identify biomarkers of nephropathy in CKD.

## 1. Introduction

Extracellular vesicles (EVs) correspond to a heterogeneous group of biological membrane-derived particles. The EVs can be classified according to their biogenesis, origin, biological function, or content [1]. EVs are subdivided into apoptotic bodies, microvesicles, and exosomes. In all three subtypes of EVs, the contents (mostly proteins, RNA, or cellular debris) are surrounded by a lipid bilayer membrane. The genesis of EVs involves various mechanisms, and EVs exhibit different sizes and flotation densities [2]. Apoptotic bodies are generally the largest EVs (ranging in size from 50 nm to 5000 nm) and are released by apoptotic cells. These bodies have an important role in immune regulation, the activation of phagocytosis pathways, and the elimination of dead cells [3]. Exosomes (50 nm to 150 nm in size) and microvesicles (100 nm to 1000 nm in size) are typically secreted by cells as a means of maintaining communication with other cells [4]. Unlike apoptotic bodies (produced only at a specific point in the cell’s life cycle), microvesicles and exosomes are produced throughout the life cycle in response to physiological and/or pathological extracellular stimuli. The lipid composition of microvesicles is closer to that of the parent cell membrane [1]. It is often difficult to distinguish precisely between exosomes and microvesicles, and so the general designation “extracellular vesicles” is sometimes preferred [5].

In the healthy kidney, EVs mediate intercellular communication and cell homeostasis, both of which are involved in electrolyte/water balance, tubular cell regeneration, and inflammatory reactions [6]. EVs appear to have an important role in kidney diseases that cause glomerular and/or tubular damage and that may ultimately lead to fibrosis. Indeed, in response to glomerular injury, damaged podocytes release EVs; in turn, the EVs may be internalized by tubular epithelial cells, where they induce apoptosis [7]. This finding suggests that the development of tubulointerstitial fibrosis related to diabetes mellitus results from glomerular injury, as a starting point for a release of EVs that are toxic for the tubules and the interstitium [8]. In the context of acute kidney injury, ischaemia–reperfusion damage and hypoxia induce greater EV production by tubular epithelial cells [9].

Compared to healthy volunteers (HVs), we reasoned that (i) EVs in urine (mainly derived from renal and infiltrating cells) might provide information on kidney injuries and the underlying nephropathy in patients with chronic kidney disease (CKD), and (ii) EVs in plasma (mainly derived from endothelial cells, platelets, and immune cells) might reflect the vascular and immunological features of kidney transplant (KT) recipients.

The objective of the present study was to identify a marker of nephropathy in patients with CKD and a marker of rejection in KT recipients by assessing EVs derived from the glomerulus (podocalyxin^+^), the proximal tubules (aquaporin-1^+^), the endothelium (CD144^+^), the immune system (CD19^+^ B-cells, CD3^+^ T-cells, CD16^+^ and CD56^+^ Natural Killer cells) and the renal progenitors (CD133^+^).

## 2. Results

### 2.1. Characteristics of the Study Population

**Figure 1:**
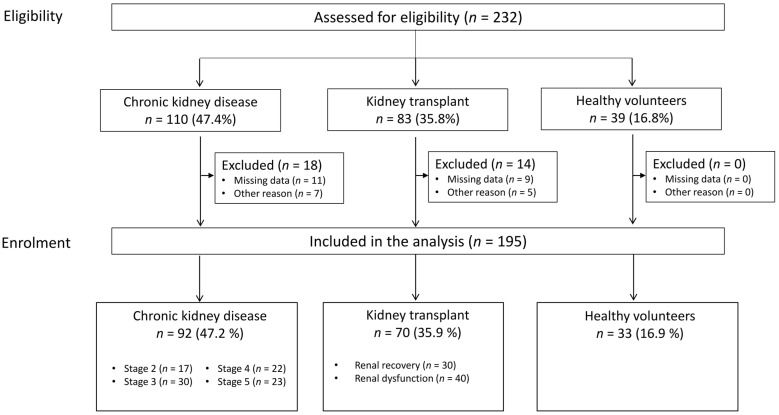
Study flow chart.

We analyzed a total of 195 individuals: 92 patients with CKD, 70 KT recipients, and 33 HVs (Figure 1). At baseline, patients with CKD were older, exerted a higher prevalence of diabetic nephropathy, and had a lower estimated glomerular filtration rate (eGFR) and higher proteinuria values (Table 1). The most common nephropathy was glomerulopathy in CKD and KT recipients and polycystic kidney disease in KT recipients. The patients with CKD and the KT recipients did not differ significantly with regard to haematological variables.

In the CKD group, there were 17 stage II patients, 30 stage III patients, 22 stage IV patients, and 23 stage V patients (Supplemental Table S1). Advanced CKD was associated with a lower haemoglobin concentration and a higher proteinuria value. The CKD stage subgroups did not differ significantly with regard to the type of nephropathy.

Among the 70 KT recipients, 30 had good allograft function (i.e., renal recovery), and 40 had renal dysfunction (including 13 with antibody-mediated rejection (ABMR), 12 with T-cell-mediated rejection (TCMR) or borderline rejection, and 15 with allograft dysfunction (AD) (Supplemental Table S2)). KT recipients presenting with rejection or AD showed greater impairments in kidney function and more severe anaemia. Proteinuria values were especially high in patients with ABMR.

### 2.2. Quantification of Plasma and Urine EV Concentrations

We analyzed a total of 390 samples (195 serum samples and 195 urine samples) from HVs, patients with CKD, and KT recipients (Figure 1). Urine EV levels were low in patients with CKD, and plasma EV levels were high in KT recipients (Table 2).

**Figure 2:**
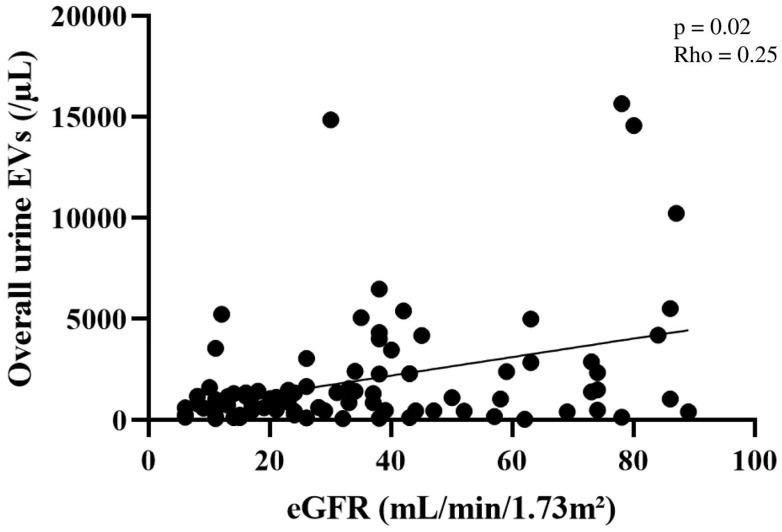
The total urine EV concentration as a function of the eGFR in the CKD group (*n* = 92). In patients with CKD, the total urine EV concentration was significantly and positively correlated with the eGFR. Each point represents the EV concentration for one CKD patient. The straight line represents the linear regression between EV concentration and eGFR. EV: extracellular vesicle, eGFR: estimated glomerular filtration rate, CKD: chronic kidney disease.

In patients with CKD, the urine EV concentration was significantly and positively correlated with the eGFR (Rho = 0.25, *p* = 0.02) (Figure 2). However, the CKD subgroups did not differ significantly with regard to the plasma EV concentration across CKD stages.

In KT recipients, regarding the numbers of plasma EVs, we detected higher levels in our KT population with renal recovery compared to the KT population with rejection or AD.

### 2.3. Characterization of Surface Antigens on Plasma and Urine EVs

The HVs, patients with CKD, and KT recipients differed with regard to the concentrations of urine and plasma EV subpopulations, with the exception of plasma CD56^+^ and CD41^+^ EVs (Table 2).

In patients with CKD, the urine concentrations of EVs produced by the renal parenchyma (podocalyxin^+^ glomerular EVs and aquaporin-1^+^ tubular EVs) and CD133^+^ EVs produced by renal progenitor cells were significantly higher in patients with CKD and an eGFR < 30 mL/min (Supplemental Table S3). By contrast, plasma subpopulation EV levels did not differ when comparing the other CKD subgroups. We did not observe any significant relationships between specific markers of glomerular and vascular nephropathies and the concentrations of glomerular or endothelial EV When a ratio is calculated between the quantity of urine EVs (total or subpopulations) on creatininuria, albuminuria, or proteinuria, the results obtained show no difference between healthy controls and patients, regardless of the stage of CKD.

**Figure 3:**
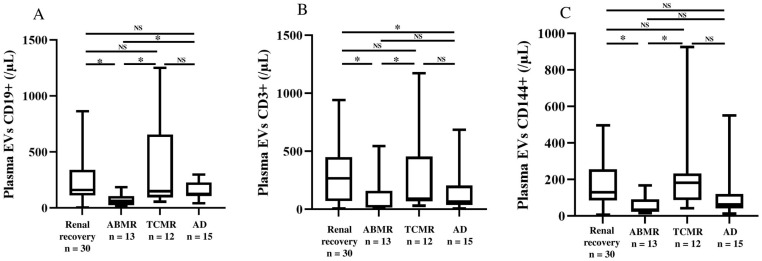
Plasma levels of EVs derived from B-cells (**A**), T-cells (**B**), and endothelial cells (**C**) in KT recipients. KT recipients with ABMR had low plasma levels of EVs derived from B-cells (CD19^+^), T-cells (CD3^+^), and endothelial cells (CD144^+^). EV: extracellular vesicle, ABMR: antibody-mediated rejection, TCMR: T-cell-mediated rejection, AD: allograft dysfunction. *: *p* < 0.05, NS: not significant.

Urine concentrations of EVs derived from endothelial cells (CD144^+^) were elevated in KT recipients with TCMR (Supplemental Table S4). Interestingly, median [IQR] urine concentrations of EVs derived from T-cells (CD3^+^) and tubular cells (aquaporin-1^+^) were higher in KT recipients with TCMR than in KT recipients with ABMR, AD, or renal recovery (respectively, 365 [185–767], 244 [75–310], 241 [113–402], and 354 [235–855]/μL; *p* = 0.06; and 1751 [357–2051], 681 [283–1016], 509 [218–919], and 827 [355–1236]/μL; *p* = 0.15), although these differences were not statistically significant. Furthermore, the plasma concentration of CD56^+^ EVs was elevated in KT recipients with TCMR. Plasma concentrations of EVs derived from B-cells (CD19^+^), T-cells (CD3^+^), and endothelial cells (CD144^+^) were lower in KT recipients with ABMR (Figure 3).

## 3. Discussion

Here, we reported on the use of a standardized, multiplex flow cytometric assay to characterize plasma and urine EVs in a cohort of patients with CKD and KT recipients.

Although we observed a relative decrease in the overall urine EV concentration as the CKD stage increased, specific subpopulations of EVs originating from the glomeruli, tubules, or vessels did not appear to be biomarkers of glomerulopathy, tubulopathy, or vascular nephropathy. Moreover, these differences in urine EV concentration according to the CKD stages were deleted when we considered EV values as a corrected ratio with creatininuria, albuminuria, and proteinuria. In this context, urinary EV levels not only reflect a specific pathogenic process but also increased overall capillary wall permeability.

The plasma EV concentrations did not depend on the CKD stage or the type of nephropathy. The urine EV signature was particularly characterized by glomerular, tubular, endothelial, and progenitor cells, which might reflect the impairment in kidney function.

The mechanism underlying the decrease in the level of urine EVs in patients with advanced CKD has not yet been characterized. Other researchers have showed that patients with CKD have significantly low urine concentrations of aquaporin-positive EVs [10]. In the kidneys, aquaporins are crucial for regulating the water balance because they facilitate the reabsorption of water from the renal tubule filtrate into the bloodstream. Specifically, the aquaporin-2 channels located in the collecting ducts are regulated by the hormone vasopressin, which increases their permeability to water; this leads to concentration of the urine, and conserves body water [11]. Experiments in rat models have shown that the release of aquaporin-positive EVs is proportional to renal levels of aquaporin expression [12,13], and that the urine level of aquaporin-positive exosomes falls during the transition from acute kidney injury to CKD [12]. These findings suggest that the low urine level of aquaporin-positive EVs in patients with CKD is due to a reduction in their expression in the kidney. In the healthy kidney, vesicles derived from the glomeruli are constantly released into the urine. An increase in the concentration of a podocyte marker in urine EVs has therefore been regarded as a direct sign of injury, whereas a reduction in the urine EV concentration might reflect a general loss of podocyte cell components in the context of chronic injury [14]. It has also been shown that patients with end-stage renal disease have low urine levels of CD133-positive EVs; this possibly indicates that these vesicles are only released by healthy renal tissue [15]. By contrast, the urine exosome level and aquaporin expression were higher in patients with diabetic nephropathy than in healthy controls [16]. Furthermore, the levels of exosome excretion were significantly higher in IgA nephropathy patients than in controls [17]. These heterogeneous findings highlight the complex mechanism(s) by which the biogenesis of urine EVs is regulated. This variability suggests that impairments in kidney function and the underlying nephropathy influence urine EV secretion differentially and independently.

Various EV subpopulations, particularly those in plasma, have been described in the literature. The plasma levels of phosphatidylserine-rich EVs are high in patients with proteinuria, hyperuricemia, and kidney function impairment due to diabetic nephropathy when compared with healthy controls [18,19]. Several interacting factors (such as uremic toxins) appear to stimulate EV release in CKD [20]. EVs are phenotypic markers of cell stress and cell activation in CKD. Interestingly, we did not observe differences in the plasma EV concentration between CKD stages. In our cohort, 22.8% of the patients with CKD had diabetic nephropathy, which is characterized by high proteinuria values. However, the diabetic nephropathy was diagnosed clinically and was often not confirmed by a kidney biopsy.

In samples from KT recipients, plasma levels of EVs derived from B-cells, T-cells, and endothelial cells were lower in KT recipients with ABMR than in KT recipients with TCMR or renal recovery. Interestingly, the difference between KT recipients with ABMR and those with AD was significant only for plasma CD19^+^ EVs.

In contrast to our results for urine EVs, plasma levels of EV subpopulations were higher in KT recipients with renal recovery than in HVs. However, these results depend on the time of sampling and the clinical event. In kidney transplant, increased circulating exosome levels are associated with acute rejection, but circulating exosomes are rapidly decreased after treatment rejection in recipients with negative peritubular capillaritis C4d, but the decrease is slower in those with positive peritubular capillaritis C4d [21].

Interestingly, plasma levels of EVs (and especially CD19^+^, CD3^+^, and CD144^+^ EVs) were much lower in KT recipients with ABMR or AD than in KT recipients with renal recovery. It is noteworthy that the plasma level of CD19^+^ EVs was the only variable specifically associated with ABMR. Earlier studies have demonstrated the presence of donor-derived human leukocyte antigen class I^+^ EVs in the circulation of KT recipients using imaging flow cytometry. After transplantation, circulating donor-derived EVs were detected in the plasma of individuals with stable allograft function but not in the plasma of individuals with AD [22]. Moreover, the plasma level of C4d^+^ endothelial EVs was elevated in patients with acute ABMR [23]. However, urine might be a better source of EVs than blood because of the limited passage of circulating EVs into the urine; this specifically increases the abundance of EVs released from glomeruli or tubular epithelial cells [24]. Some studies have evidenced differences in EV levels in KT recipients. Indeed, a high urine level of T-cell-derived EVs (CD3^+^) was observed in patients with graft rejection [25]. As in patients with end-stage renal disease, the urine level of CD133^+^ EVs is low in KT recipients with slow graft function and vascular damage [17]. The urine levels of mesenchymal progenitor cell markers (CD1c, CD105, CD133, and SSEEA-4) rise progressively post transplant in patients having achieved renal recovery [26].

The level and the composition of EVs might also have a role in the pathophysiology of cardiovascular complications [27]. Along with the EVs’ cellular origin, their composition is a crucial variable for investigation. By transporting functional proteins, lipids, metabolites, and nucleic acids (DNA, RNA, and other noncoding RNA molecules), EVs have a crucial role as mediators of intercellular communication between different cell types. For example, neutrophil gelatinase-associated lipocalin (NGAL) in urine EVs could be used as a biomarker of kidney graft function [28]. Sigdel et al. predicted acute rejection on the basis of the target proteins present in urinary exosomes but not in urine [29]. The discovery of RNA inside EVs was a milestone in demonstrating the vesicles’ role in the intercellular transfer of genetically encoded messages. Further studies of the composition of EVs are clearly warranted.

## 4. Materials and Methods

### 4.1. Patient Recruitment

All the eligible patients were receiving regular medical care in our centre for their CKD or after kidney transplantation. We screened patients with CKD and KT recipients referred in the nephrology department at Amiens University Hospital (Amiens, France) from 1 January 2021 to 31 December 2022. Patients who gave informed consent were included in the prospective analysis (Figure 1). The healthy volunteers (HVs) were not taking medications, not using tobacco, and not knowingly suffering from an acute or chronic medical condition.

The study protocols for the analysis of EVs in patients with CKD and in KT recipients were approved by an independent ethics committee called CPP Nord Ouest (NCT04700631; identifiers: ID-RCB 2020-A02352-37 and ID-RCB 2020-A033340-39, respectively). All procedures were implemented in accordance with the institution’s ethical standards and the tenets of the 1975 Declaration of Helsinki.

With regard to CKD, the disease stage was defined in accordance with the 2024 Kidney Disease Improving Global Outcomes guidelines 2024 [30]. The type of nephropathy (classified as vascular, glomerular, tubulointerstitial, diabetes, polycystic kidney disease, undetermined, or other) was documented by reference to the patients’ medical records.

With regard to KT recipients, pre-existing nephropathy was documented by reference to the patients’ medical records. We considered KT recipients to have achieved “renal function recovery” when the estimated glomerular filtration rate (eGFR) was 60 mL/min/1.73 m^2^ or more at least one year after transplantation, with no history of rejection. KT recipients having undergone a graft biopsy (due to impaired kidney function or the presence of de novo donor-specific antibodies) were classified as having “allograft rejection” or “allograft dysfunction” (AD). Graft rejections were confirmed by a biopsy and met the 2019 Banff Classification’s criteria. The 2019 Banff Classification was also used to determine the type of rejection.

### 4.2. Sampling Strategy

Blood and urine samples were collected for routine laboratory tests. Based on the serum creatinine concentration, the eGFR (expressed in mL/min/1.73 m^2^) was calculated according to the Chronic Kidney Disease Epidemiology Collaboration equation. Several standard haematological and biochemical variables were measured as part of the scheduled workup. Whenever possible, additional peripheral blood samples (12 mL in citrated tubes) and fresh urine samples (20 mL of the first morning urine) were collected under fasting conditions. The sample handling, preprocessing, and storage complied with the guidelines issued by the International Society for Extracellular Vesicles [5].

### 4.3. EV Characterization

We quantified the urine and plasma EV levels in patients with CKD and in KT recipients as previously described [31]. We described subpopulations of annexin V-positive EVs originating from:

- the glomerulus (podocalyxin^+^), proximal tubules (aquaporin-1^+^), endothelium (CD144^+^), and platelets (CD41^+^) to localize the pathologic process;
- the immune system B-cells (CD19^+^), T-cells (CD3^+^), and natural killer cells (CD16^+^ and CD56^+^) to highlight the immune mechanism of the kidney injury;
- the renal progenitors (CD133^+^) to assess the renal recovery.

Serum and urine samples were characterized by a multiplex flow cytometry assay. Blood EVs were pelleted by centrifugation (25,000× *g*, 30 min) from platelet-poor plasma. For urine samples, a first centrifugation (500× *g*, 15 min) was performed to remove cells and debris, followed by treatment with dithiothreitol (0.05 M, 25 µL/mL, 10 min at +37 °C) to lyse uromodulin protein, and then EVs were pelleted by centrifugation (25,000× *g*, 30 min). EV-enriched pellets were resuspended in PBS and stored at −80 °C, and for analysis, samples were rapidly thawed at 37 °C. EV detection was then performed on thawed samples using a Cytoflex cytometer (Beckman Coulter Life Sciences, Villepinte, France). The following antibodies were used for EV detection: BV510-conjugated annexin V as the EV marker (BioLegend, Paris, France), phycoerythrin (PE)-conjugated anti-podocalyxin (ThermoFisher Scientific, Waltham, MA, USA) as the podocyte marker, fluorescein (FITC)-conjugated anti-aquaporin 1 (Clinisciences, Nanterre, France) as the proximal tubule marker, allophycocyanin (APC)-conjugated anti-CD133 (Beckman Coulter Life Sciences, Villepinte, France) as the progenitor cell marker, and PE-conjugated anti-CD144-PE as the endothelial marker (Beckman Coulter Life Sciences, Villepinte, France). Megamix-plus FSC beads (BioCytex, Marseille, France) calibrated from 0.1 to 0.9 µm were used to define an analysis window consistent with the size of EVs. EVs were quantified using EV Count Beads (BioCytex, Marseille, France): (EV counts × [EV Count beads])/EV Count beads counted. In order to discriminate EVs and aggregates, we set the signal detection at peak height (V-SSC-H and FSC-H) and peak width.

### 4.4. Statistics

Categorical variables were expressed as the frequency (percentage), and continuous variables were presented as the median [interquartile range (IQR)]. Fisher’s exact test was applied to compare frequencies, while Mann–Whitney and Kruskal–Wallis nonparametric tests were used to compare median [IQR] values from two or more groups. Spearman’s correlation coefficient was calculated as a guide to the strength of the correlation between two continuous variables. The threshold for statistical significance was set to *p* < 0.05. Statistical analyses were performed using R software (version 4.2.3) via the RStudio Inc., Vienna, Austria, interface (version 2022.12.0). Figures were generated using GraphPad Prism software (version 10.2.0, GraphPad Software LLC, Boston, MA, USA).

## 5. Conclusions

We analyzed EVs in matched serum and urine samples from patients with CKD and from KT recipients. In CKD, the urine EV level fell and the CKD stage increased, although no specific biomarkers of nephropathy were apparent. In KT recipients with allograft disease, low plasma levels of CD19^+^, CD3^+^, and CD144^+^ EVs were correlated with ABMR and AD. Although a low plasma level of CD19^+^ EVs appeared to be specific for ABMR, this potential biomarker requires further investigation.

## Figures and Tables

**Table 1:** Demographic, clinical, and biochemical characteristics of HVs, patients with CKD, and KT recipients.

Variables	HVs *n* = 33	Patients with CKD *n* = 92	KT Recipients *n* = 70	*p*
**Clinical Variables**				
Age, years, median [range]	29 [26–48]	66 [54–73]	55 [49–65]	<0.001
Male sex, *n* (%)	19 (57.6)	44 (47.8)	44 (62.8)	0.16
BMI, kg/m^2^, median [range]	21.9 [20.7–23.2]	27.5 [25.0–31.1]	25.9 [23.5–28.4]	<0.001
***Nephropathies, n (%)***				
Vascular	-	8 (8.7)	6 (8.6)	1
Diabetes	-	21 (22.8)	3 (4.3)	<0.001
Glomerulonephritis	-	37 (40.2)	27 (38.6)	0.87
Tubulointerstitial nephritis	-	11 (11.9)	16 (22.9)	0.09
Polycystic kidney disease	-	3 (3.3)	9 (12.8)	0.03
Undetermined	-	7 (7.6)	6 (8.6)	1
Other	-	5 (5.4)	3 (4.3)	1
**Biochemical Variables—Median [Range]**				
***In the plasma***				
Serum creatinine, µmol/L	-	176 [118–296]	113 [91–169]	<0.001
eGFR, mL/min/1.73 m^2^	-	30.5 [15.7–45.5]	60.5 [35.2–73.0]	<0.001
Haemoglobin, g/dL	-	12.3 [10.9–13.9]	13.0 [11.7–14.3]	0.13
White cell count, G/L	-	7.3 [5.7–9.1]	7.3 [6.0–8.9]	0.91
Platelet count, G/L	-	242 [208–289]	230 [191–275]	0.17
**In the urine**				
Protein, mg/L	-	681 [140–1507]	179 [104–442]	<0.001
Albumin, mg/L	-	182 [23–859]	26 [8–139]	<0.001
Creatinine, mmol/L	-	5.7 [3.8–8.0]	6.2 [4.5–8.7]	0.15

HV: healthy volunteer, CKD: chronic kidney disease, KT: kidney transplant, BMI: body mass index, eGFR: estimated glomerular filtration rate. Data are expressed as the median [range] or *n* (%).

**Table 2:** The median [range] urine and plasma EV concentrations in healthy volunteers, patients with CKD, and kidney transplant recipients.

Variables	HVs *n* = 33	Patients with CKD *n* = 92	KT Recipients *n* = 70	*p*
**Urine**				
Overall	2486 [1330–4746]	1045 [464–2323]	1875 [946–3506]	<0.001
Podocalyxin	1438 [837–2202]	398 [173–845]	1154 [321–1998]	<0.001
Aquaporin-1	1053 [686–1492]	362 [171–725]	757 [288–1441]	<0.001
CD133	582 [377–851]	118 [49–290]	298 [118–597]	<0.001
CD144	843 [315–1008]	198 [55–433]	509 [194–1022]	<0.001
CD19	455 [175–683]	151 [55–432]	358 [190–807]	<0.001
CD3	458 [213–783]	105 [43–283]	304 [148–680]	<0.001
CD16	614 [329–1063]	195 [75–480]	450 [174–1107]	<0.001
CD56	246 [145–470]	71 [34–147]	170 [66–328]	<0.001
CD41	399 [185–678]	160 [66–381]	394 [202–791]	<0.001
**Plasma**				
Overall	1402 [878–1878]	1171 [549–2082]	2620 [1062–4272]	<0.001
Podocalyxin	116 [77–266]	111 [41–226]	465 [100–956]	<0.001
Aquaporin-1	154 [95–287]	175 [70–422]	685 [179–1062]	<0.001
CD133	undetected	undetected	undetected	-
CD144	43 [22–67]	41 [21–85]	107 [43–200]	<0.001
CD19	102 [65–169]	80 [48–178]	135 [86–228]	0.02
CD3	52 [36–98]	48 [22–85]	130 [39–355]	<0.001
CD16	135 [72–207]	76 [34–217]	202 [73–489]	0.008
CD56	29 [17–52]	24 [14–39]	20 [12–39]	0.27
CD41	1197 [649–1873]	902 [318–1673]	1287 [348–2211]	0.35

HV: healthy volunteers, CKD: chronic kidney disease, KT: kidney transplants, EV: extracellular vesicle. The EV concentration is expressed in EVs/µL. Data are expressed as the median [range].

## Data Availability

The data presented in this study are available on request from the corresponding author.

## Supplementary Materials

The supporting information can be downloaded at https://www.mdpi.com/article/10.3390/ijms26083635/s1.
