# Supplementary material for: Quantification of Urine and Plasma Levels of Extracellular Vesicles in a Cohort of Kidney Transplant Recipients and Chronic Kidney Disease Patients
Source: Int J Mol Sci. 2025 Apr 11;26(8):3635. doi: 10.3390/ijms26083635 (PMC12027010; doi:10.3390/ijms26083635)
Supplement: Supplementary file 1 [file ijms-26-03635-s001.zip › ijms-3542910-supplementary.pdf]

Supplemental table 1: Demographic, clinical and biochemical characteristics of CKD patients.

Variables	Stage II CKD n=17	Stage III CKD n=30	Stage IV CKD n=22	Stage V CKD n=23	p
**Clinical variables**					
Age, years median [range]	59 [51-71]	67 [60-73]	65 [55-77]	70 [51-73]	0.36
Male sex, n (%)	9 (52.9)	15 (50.0)	8 (33.3)	12 (57.1)	0.40
BMI, kg/m² median [range]	26.3 [23.1-30.1]	28.5 [26.2-32.2]	27.8 [25.7-31.1]	26.5 [23.8-30.8]	0.26
Nephropathies, n (%)					
Vascular	3 (18.0)	3 (10.0)	1 (4.0)	1 (4.0)	0.48
Diabetes	1 (6.0)	6 (20.0)	8 (33.0)	6 (29.0)	0.17
Glomerulonephritis	7 (41.0)	12 (40.0)	10 (42.0)	8 (38.0)	1
Tubulo-interstitial nephritis	2 (12.0)	6 (20.0)	1 (4.0)	2 (10.0)	0.38
Polycystic kidney disease	1 (6.0)	0 (0.0)	1 (4.0)	1 (5.0)	0.54
Undetermined	2 (12.0)	2 (7.0)	0 (0.0)	3 (14.0)	0.24
Other	1 (6.0)	1 (3.0)	3 (12.0)	0 (0.0)	0.33
**Biochemical variables**					
In the plasma					
Serum creatinine, µmol/L	88 [76-95]	140 [129-149]	224 [188-282]	396 [356-590]	< 0.001
eGFR, mL/min/1.73m²	74 [73-84]	38 [35-44]	21 [17-24]	11 [8-13]	< 0.001
Haemoglobin, g/dL	13.9 [13.2-14.3]	13.2 [11.9-14.6]	11.5 [10.9-12.5]	10.6 [9.8-11.7]	< 0.001
White cell count, G/L	6.3 [5.6-7.6]	8.0 [6.0-9.6]	7.3 [6.3-7.6]	8.2 [5.7-9.4]	0.37
Platelet count, G/L	245 [217-278]	247 [221-289]	240 [196-286]	221 [191-289]	0.62
In the urine					
Protein, mg/L	210 [98-627]	313 [98-799]	696 [247-1941]	1283 [828-1818]	0.009
Albumin, mg/L	29 [19-402]	45 [7-371]	269 [53-1140]	569 [344-1076]	0.009
Creatinine, mmol/L	9.5 [6.3-10.9]	6.6 [4.4-9.1]	4.0 [2.2-6.7]	4.6 [3.6-5.6]	0.001

BMI: body mass index, eGFR: estimated glomerular filtration rate

Data are expressed as the median [range] or n (%)

Supplemental table 2: Clinical and biochemical characteristics of kidney transplant recipients.

Variables	Renal recovery n=30	ABMR n=13	TCMR/borderline n=12	AD n=15	p
**Clinical variables**					
Age, years	56 [50-68]	52 [50-61]	56 [42-64]	57 [49-66]	0.68
Male sex, n (%)	22 (73.3)	7 (53.8)	7 (46.7)	8 (66.7)	0.30
BMI, kg/m²	26.4 [23.2-29.2]	27.5 [24.9-29.1]	23.8 [22.9-24.2]	26.0 [24.6-27.3]	0.10
Nephropathies, n (%)					
Vascular	3 (10.0)	1 (7.7)	0 (0.0)	2 (16.7)	0.47
Diabetes	2 (6.7)	0 (0.0)	1 (6.7)	0 (0.0)	1
Glomerulonephritis	13 (43.3)	5 (38.5)	4 (26.7)	5 (41.7)	0.76
Tubulo-interstitial nephritis	6 (20.0)	3 (23.1)	6 (40.0)	1 (8.3)	0.28
Polycystic kidney disease	5 (16.7)	0 (0.0)	3 (20.0)	1 (8.3)	0.39
Undetermined	1 (3.3)	3 (23.1)	1 (6.7)	1 (8.3)	0.16
Other	0 (0.0)	1 (7.7)	0 (0.0)	2 (16.7)	0.03
**Biochemical variables**					
In the plasma					
Serum creatinine, µmol/L	91 [77-99]	171 [123-216]	162 [136-169]	165 [143-190]	<0.001
eGFR, mL/min/1.73m²	75.5 [67.2-82.0]	32.0 [30.0-53.0]	43.0 [36.0-50.2]	35.0 [26.5-45.0]	< 0.001
Haemoglobin, g/dL	14.0 [13.0-15.0]	11.2 [9.7-12.5]	12.3 [11.7-13.4]	11.7 [10.9-13.6]	< 0.001
White cell count, G/L	7.3 [6.4-8.7]	7.0 [5.2-8.3]	7.6 [5.1-9.9]	7.0 [6.3-9.0]	0.90
Platelet count, G/L	232 [194-293]	195 [175-238]	246 [215-268]	227 [187-272]	0.35
In the urine					
Protein, mg/L	116 [79-142]	761 [210-1082]	243 [140-340]	280 [122-924]	<0.001
Albumin, mg/L	8 [5-26]	228 [66-533]	35 [12-64]	74 [14-427]	<0.001
Creatinine, mmol/L	5.8 [4.5-8.4]	5.9 [3.2-6.7]	8.1 [5.7-11.4]	7.1 [6.0-12.2]	0.05

ABMR: antibody-mediated rejection, TCMR: T-cell mediated rejection, AD: allograft dysfunction, BMI: body mass index, eGFR: estimated glomerular filtration rate

Supplemental table 3: Urine and plasma levels of EVs, by CKD stage.

EV levels are expressed in EVs/µL.

Variables	Stage 2 n=17	Stage 3 n=30	Stage 4 n=22	Stage 5 n=23	p
**Urine**					
Total	2347 [497-4990]	1335 [466-3195]	689 [487-1299]	681 [239-1174]	0.06
Podocalyxin	970 [365-2719]	446 [196-1283]	316 [200-408]	200 [81-417]	0.002
Aquaporin-1	791 [294-1290]	433 [194-904]	288 [175-518]	211 [97-557]	0.04
CD133	321 [161-781]	165 [51-471]	58 [50-118]	68 [20-147]	0.002
CD144	407 [117-891]	174 [64-536]	162 [69-242]	119 [42-291]	0.10
CD19	350 [121-592]	123 [43-316]	141 [83-254]	150 [48-423]	0.18
CD3	286 [71-528]	133 [44-423]	83 [42-138]	85 [43-235]	0.06
CD16	477 [258-816]	161 [49-580]	179 [133-275]	145 [45-322]	0.10
CD56	248 [125-340]	57 [25-132]	78 [46-102]	53 [20-114]	0.008
CD41	366 [238-630]	101 [51-254]	157 [98-334]	186 [67-319]	0.05
**Plasma**					
Total	722 [302-1817]	1089 [903-1817]	1132 [525-2437]	1322 [565-1864]	0.74
Podocalyxin	55 [36-146]	111 [56-324]	103 [36-251]	129 [72-165]	0.64
Aquaporin-1	73 [53-130]	212 [108-370]	150 [66-637]	212 [76-488]	0.18
CD133	Not detected	Not detected	Not detected	Not detected	-
CD144	32 [19-56]	40 [16-59]	55 [23-107]	60 [24-123]	0.57
CD19	95 [73-163]	84 [56-107]	73 [38-384]	76 [42-272]	0.95
CD3	44 [20-92]	56 [21-76]	57 [27-96]	47 [25-77]	0.91
CD16	28 [18-56]	68 [53-234]	134 [27-510]	73 [48-172]	0.15
CD56	31 [11-53]	24 [13-33]	22 [13-37]	30 [20-47]	0.51
CD41	222 [131-1461]	1071 [603-1407]	880 [333-2102]	1020 [410-1698]	0.5

Supplemental table 4: Clinical and biochemical characteristics of KT recipients

Variables	Renal recovery n=30	ABMR n=13	TCMR n=12	AD n=15	p
**Urine**					
Overall	2334 [1138-3936]	1575 [1052-2327]	2210 [742-4517]	1423 [848-3197]	0.29
Podocalyxin	1254 [411-2183]	1054 [262-1404]	2412 [434-2929]	579 [336-1332]	0.28
Aquaporin-1	827 [355-1236]	681 [283-1016]	1751 [358-2051]	509 [218-919]	0.15
CD133	378 [151-648]	243 [41-352]	445 [170-946]	149 [113-462]	0.10
CD144	546 [365-1159]	294 [133-610]	1002 [217-1552]	311 [119-782]	0.05
CD19	459 [221-745]	220 [164-528]	741 [218-1179]	207 [118-622]	0.16
CD3	354 [235-855]	244 [75-310]	365 [185-767]	241 [113-402]	0.06
CD16	580 [190-1235]	228 [144-505]	796 [260-1838]	447 [249-1082]	0.55
CD56	231 [101-453]	99 [62-135]	252 [76-444]	114 [69-239]	0.16
CD41	372 [153-747]	255 [178-455]	538 [223-1817]	594 [287-963]	0.96
**Plasma**					
Overall	4058 [2893-5796]	1097 [695-2148]	2603 [1609-4541]	1217 [416-2720]	< 0.001
Podocalyxin	607 [439-1398]	238 [75-523]	444 [145-1134]	323 [59-727]	0.08
Aquaporin-1	1013 [563-1455]	200 [70-452]	671 [288-854]	498 [113-861]	0.02
CD133	Not detected	Not detected	Not detected	Not detected	-
CD144	130 [99-235]	33 [24-69]	182 [100-230]	64 [48-117]	0.005
CD19	160 [111-316]	62 [41-98]	149 [103-631]	124 [106-226]	0.003
CD3	266 [74-414]	26 [20-101]	92 [90-264]	67 [39-175]	0.009
CD16	289 [106-612]	220 [61-299]	145 [105-366]	79 [50-406]	0.20
CD56	21 [9-39]	12 [10-15]	56 [35-91]	22 [12-35]	0.009
CD41	1849 [1257-4799]	426 [245-1481]	807 [457-2413]	329 [204-1271]	< 0.001

ABMR: antibody-mediated rejection, TCMR: T-cell mediated rejection, AD: allograft dysfunction, BMI: body mass index, eGFR: estimated glomerular filtration rate
